# Onions

**Published:** 1858-04

**Authors:** 


### ONIONS

ARE one of the most nutritious, healthful, and detestable articles of food found in our markets. We never ate one to our knowledge, and never expect to; we can smell them a mile off, perhaps. A few grains of roasted coffee, eaten immediately afterwards, or a teaspoonful or two of vinegar swallowed, removes at once the odor from the breath. If onions are half-boiled, the water thrown away, and are then put in soup to be boiled "done," the odor will be but little noticed.

